# Comparative evaluation of bio-fertilizer replacement with chemical fertilizer in sesame (*Sesamum indicum* L) production under drought stress and normal irrigation condition

**DOI:** 10.1016/j.heliyon.2025.e42743

**Published:** 2025-02-15

**Authors:** Nasser Nourzadeh, Asghar Rahimi, Amir Dadrasi

**Affiliations:** Department of Genetic and Plant Production, Faculty of Agriculture, Vali-e-Asr University of Rafsanjan, Rafsanjan, Iran

**Keywords:** Sesame, Biostimulants, Mycorrhiza, Effect size, Drought stress

## Abstract

Drought stress represents a considerable environmental challenge, exerting a deleterious effect on plant growth and productivity. In order to address this issue, the use of biostimulants, such as plant growth-promoting rhizobacteria (PGPR) and arbuscular mycorrhizal fungi (AMF), has gained increasing attention in recent years. The present study, conducted in 2022, sought to evaluate the effects of biological and chemical fertilizers under drought-stress conditions on sesame yield and water-use efficiency. The research was conducted at two farms, Dashthouz and Sarkahnan, which are located approximately 80 km apart in Rodan city, Hormozgan province, Iran. The research was designed as a factorial experiment using a randomized complete block design (RCBD) with three replications. The study examined two main factors: fertilizer application, with eight levels (bacteria (B), mycorrhizal fungi (MY), chemical fertilizer (NPK), bacteria + mycorrhizal fungi (B+MY), bacteria + chemical fertilizer (B+NPK), mycorrhizal fungi + chemical fertilizer (MY+NPK), bacteria + mycorrhizal fungi + chemical fertilizer (B+MY+NPK), and a control), and drought stress, with two levels (normal irrigation without drought stress and drought stress). The results indicated that the main effects of location, irrigation, fertilizer application, and their interactions significantly influenced the leaf area index (LAI), number of branches, number of capsules, number of seeds per capsule, seed yield, biological yield, harvest index, oil yield, meal yield, and water-use efficiency. However, there was no significant effect on thousand-seed weight. This indicates that all measured traits were influenced by the experimental factors. Regarding seed yield, the lowest value of 95.3 g/m^2^ was recorded in the control treatment under normal irrigation conditions at Dachthouz, while the highest value of 325.5 g/m^2^ was achieved in the control treatment under normal irrigation conditions at Sarkahnan. The findings revealed that the application of mycorrhizal fungi (MY) and bacteria (B) as substitutes for phosphorus and nitrogen, respectively, produced seed yields comparable to those achieved with NPK fertilizers under normal irrigation conditions. However, under drought stress conditions, water scarcity disrupted the symbiotic interactions between the microorganisms and the crop, reducing the effectiveness of MY and B treatments in enhancing crop growth and yield. These results contribute to advancing sustainable sesame production systems by minimizing the reliance on chemical fertilizers and enhancing crop resilience to drought stress. Further research and practical implementation of these strategies could lead to more efficient and environmentally sustainable sesame cultivation practices.

## 1 Introduction

Sesame (*Sesamum indicum* L.) is one of the world's most important oilseed crops, recognized for its high-quality oil and nutritionally rich seeds, which contribute significantly to both the food and industrial sectors [1]. It is cultivated in various regions, including those characterized by arid and semi-arid climates, where its production is limited by water scarcity and drought stress [2]. These environmental challenges are expected to intensify due to climate change, leading to more frequent and prolonged periods of water stress that significantly impact crop productivity and sustainability [3]. Drought stress, particularly in water-scarce regions, adversely affects plants by reducing photosynthesis, respiration, and overall crop yield, which ultimately threatens food security and agricultural viability [4].Water scarcity and the subsequent drought stress disrupt critical physiological processes in plants, such as stomatal closure, reduced net photosynthesis, and diminished transpiration, all of which result in reduced water potential and a decrease in plant vitality [6]. Additionally, drought stess induces the accumulation of reactive oxygen species (ROS), which cause oxidative damage to cellular macromolecules, membranes, and lipids, exacerbating the stress [4,5]. In response, plants enhance their antioxidant defense systems, increasing the activity of enzymes such as catalase and peroxidase to mitigate cellular damage and maintain homeostasis [7]. Consequently, mitigating the impacts of water stress has become a critical research focus, with the goal of improving crop resilience and productivity under increasingly unpredictable climatic conditions [6].

In the pursuit of sustainable agricultural practices, one promising strategy is the implementation of regulated deficit irrigation (RDI), a practice where water application is reduced below the crop's optimal requirements, but not so severely as to compromise crop survival [6]. This technique has proven effective in conserving water while maintaining reasonable crop yields, particularly in regions suffering from chronic water shortages. However, alongside water-saving irrigation strategies, enhancing soil fertility and plant health through sustainable fertilization practices is equally crucial [8]. The conventional reliance on chemical fertilizers, particularly nitrogen-based compounds, to boost soil fertility has led to significant environmental issues, including soil degradation, water contamination, and adverse health effects due to the persistence of harmful chemicals in ecosystems [9]. The negative impacts of over-reliance on chemical fertilizers necessitate the exploration of alternative solutions, particularly those that promote ecological balance and reduce the environmental footprint of agricultural systems [10]. In this context, bio-fertilizers, which consist of beneficial microorganisms such as bacteria, fungi, and algae, have gained considerable attention as a sustainable alternative to chemical fertilizers. These organisms enhance nutrient availability, improve soil structure, and increase plant resistance to abiotic stresses, including drought [11]. By promoting mechanisms like nitrogen fixation, phosphate solubilization, and the production of growth-promoting substances, bio-fertilizers can enhance the resilience of crops, improve their water-use efficiency, and reduce dependency on chemical inputs [12]. Mycorrhiza, a prominent bio-fertilizer, is particularly effective in improving root development, enhancing nutrient and water uptake, and mitigating the impacts of water stress [11].

Recent studies have demonstrated the synergistic potential of combining bio-fertilizers with chemical fertilizers to achieve higher crop yields while maintaining soil health and reducing environmental damage [[12], [13], [14]]. By combining the immediate nutrient supply of chemical fertilizers with the long-term benefits of bio-fertilizers, this integrated approach offers a balanced solution that maximizes plant productivity while minimizing the ecological costs associated with chemical fertilization [15]. This synergistic effect is particularly promising for crops like sesame, which face unique challenges in water-scarce environments, as it enhances both plant growth and water-use efficiency [16,17]. Despite the substantial body of research on the impacts of water stress and the potential of bio-fertilizers to mitigate these effects, few studies have investigated the integrated application of bio- and chemical fertilizers in sesame cultivation under drought conditions [18]. This gap in the literature underscores the need for further exploration of how these two fertilization strategies can work together to improve crop resilience, water-use efficiency, and soil sustainability in arid and semi-arid regions. Therefore, the present study aims to address this knowledge gap by evaluating the combined effects of bio and chemical fertilizers under both drought stress and normal irrigation conditions, specifically focusing on sesame cultivation.The key innovation of this study lies in its comprehensive evaluation of the synergistic interactions between bio- and chemical fertilizers in optimizing sesame yield, water-use efficiency, and soil health under drought stress. Employing a factorial experimental design across two distinct locations, this study provides valuable insights into how these treatment interactions vary in different environmental contexts. Additionally, meta-analysis approaches are used to quantify the percentage change in yield, water-use efficiency, and soil health parameters compared to control treatments. By combining experimental and statistical methodologies, this research aims to contribute significantly to the development of sustainable agricultural practices that reduce dependency on chemical fertilizers while enhancing crop resilience in water-limited regions.

## 2 Material and methods

**Fig. 1:**
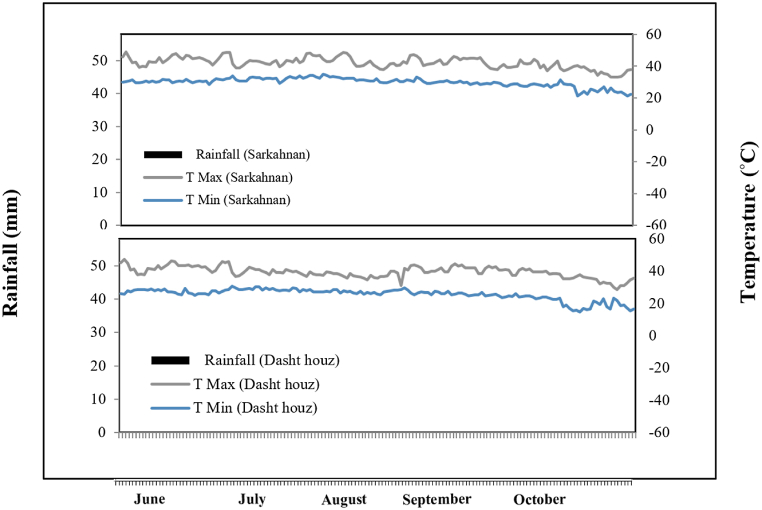
Average precipitation and temperature of the experimental sites during the growing season of 2022–2023.

This research was conducted in 2022 in two farms, Dashthouz and Sarkahnan, located approximately 80 km from each other in the Rodan city of Hormozgan province, Iran. Sarkohanan, located in the southern region of Iran within the Hormozgan province, is characterised by geographical coordinates of 27°25′ N latitude and 57°12′ E longitude. Positioned in the northeastern part of Hormozgan province, Sarkohanan shares borders with Rodan and Jask counties. The region experiences a predominantly hot and dry climate throughout most months of the year. The average summer temperature is approximately 45 °C, while in winter it is around 20 °C [19]. Dashthouz, situated in the south of Iran within the Hormozgan province, is located at geographical coordinates of 27°40′ N latitude and 57°10′ E longitude. Dashthouz is located in the northwestern part of Hormozgan province, sharing borders with Beshagard and Bastak cities. The climate of Dashthouz is characterised as hot and dry, with average summer temperatures reaching approximately 45 °C and winter temperatures averaging around 20 °C. The majority of rainfall in this region occurs during the autumn and winter seasons [19]. Prior to conducting the experiment, a composite soil sample was collected from the field at a depth of 0–30 cm. The soil sample was then subjected to rigorous analysis to ascertain its physical and chemical characteristics. The results of the soil analyses are presented in Table 1. Also, The climate parameters, Average precipitation and temperature of the experimental sites during the growing season of 2022–2023, are illustrated in Fig. 1.

**Table 1:** Physical and chemical characteristics of the Dashthouz and Sarkahnan soils.

Location	Ec (ds/m)	pH	Organic carbon (%)	P (ppm)	K (ppm)	N (%)	Clay (%)	Silt (%)	Sand (%)	Soil texture
Sarkahnan	5.41	7.59	0.64	40.5	182.32	0.05	10	22	68	Loamy Sand
Dashthouz	2.1	7.95	0.38	5.2	208.9	0.04	4	16	80	Loamy Sand

The research was conducted as a factorial experiment, employing a randomised complete block design with three replications. The experimental period encompassed the months from June to October. The study comprised two factors incloding fertilizer application with eight levels (bacteria (B), mycorrhizal fungus (MY), chemical fertilizer (NPK), bacteria + mycorrhizal fungus (B+MY), bacteria + chemical fertilizer (B+NPK), mycorrhizal fungus + chemical fertilizer (NPK+MY), bacteria + mycorrhizal fungus + chemical fertilizer (B+MY+NPK), and control) and drought stress, which had two levels: normal irrigation without drought stress and drought stress.

Based on the soil analysis test results, the recommended fertilizers application rates for the Sarkahnan region are as follows: 300 kg/ha of urea, no phosphorus application, and 200 kg/ha of potassium sulfate. For the Dashthouz region, the recommended rates are 350 kg/ha of urea, 150 kg/ha of phosphorus, and 150 kg/ha of potassium sulfate. The application of chemical fertilizers was tailored to meet the specific nutrient requirements of the plants and based on soil test results. In the treatment involving the application of pure chemical fertilizers, the application rate was determined according to the plant's needs. Conversely, the treatments involving the utilization of mycorrhizal fungi and bacteria involved a 50 % reduction in nitrogen fertilizers and a 50 % reduction in phosphorus fertilizers. This approach was adopted to enhance the efficiency of fertilizer usage, while leveraging the advantageous effects of mycorrhizal fungi and bacteria in nutrient uptake and utilization.

In the present experiment, Pseudomonas putida bacteria and a mixture of Rhizophagos intraradices and Fanliformis Mose mycorrhizal fungus were utilised. These bacterial and fungal treatments were obtained from Danesh sabz Mahan Company and Ronak Biotechnology Company and Vira Gostar Company, respectively. The application of bacteria and fungi followed the instructions provided by the respective manufacturers. The application of the mycorrhizal fungi was conducted prior to the planting stage, while the bacteria were applied following the thinning of the plants. The chemical fertilizers employed in the study included urea, triple superphosphate, and potassium sulfate, which were selected to satisfy the nitrogen, phosphorus, and potassium requirements of the plants, as determined by rigorous soil testing and a comprehensive analysis of the plant's nutritional needs. Prior to the cultivation of sesame plants, a series of land preparation activities were undertaken, including plowing, disking, and leveling. Triple superphosphate and potassium sulfate fertilizers s were applied to the designated plots according to the experimental plan and incorporated into the soil. The mycorrhizal fungi were then dispersed into the furrows created for seed planting, covered with a thin layer of soil, and followed by manual seeding of the sesame seeds. Irrigation was then administered in accordance with the experimental design.

This experimental approach enabled the examination of the combined effects of different fertilization treatments and drought stress conditions on sesame crop growth and productivity. In mid-July, the sesame plant variety "Dashtestan2" was cultivated using high-density planting techniques. The planting configuration comprised rows with a spacing of 50 cm, arranged in a bed format, and stacked vertically. The plot size was 2 × 4 m. The distance between individual plots was one and a half meters, while the gap between blocks was 2 m. Thinning of the plants was conducted when they reached the 3 to 4 leaf stage in order to achieve the desired plant density (25 plant/m^2^ approximately 200 plants per plot). The thinning process entailed the removal of excess plants, with the objective of achieving a spacing of 8 cm^2^ between plants on each row. Following this, the bacterial treatment was administered during the irrigation process. The application method involved the dissolution of 1 kg of bacteria in 1 L of water. The resulting solution was then mixed thoroughly with 100 L of water to obtain a uniform solution. This solution was then administered to the plants through the irrigation system. The application of urea fertilizers was conducted in two stages. The first application was made before flowering, when the plants had reached a height of approximately 20 cm. The second application was initiated at the onset of flowering and the initial formation of capsules. The urea fertilizers was added in liquid form, similar to vinegar.

The implementation of these cultivation practices was driven by the necessity to ensure appropriate plant spacing, to promote plant health, and to provide necessary nutrients for optimal growth and development of the sesame plants. The irrigation process was executed through the utilization of a micro-irrigation approach. The initial irrigation was administered immediately post-planting, ensuring that all treatments received adequate water until the 3–4 leaf stage of plant growth and establishment. Following this, a drought stress was initiated.

Drought stress was imposed using a Class A evaporation pan to monitor cumulative water loss. For the drought-stressed plots, irrigation was with held until 100 mm of cumulative evaporation was reached. In contrast, the control plots were irrigated at 50 mm of cumulative evaporation. The days required to reach these thresholds varied with environmental conditions. In the early growth stage, it took 5–6 days to reach 50 mm evaporation, while in the later stages, it took 3–4 days. Control plots were irrigated every 5–7 days, maintaining consistent soil moisture, whereas drought-stressed plots were irrigated every 10–12 days. Notably, as indiated in Fig. 1 no rainfall occurred during the growing season, and thus, rainfall was not a factor in irrigation scheduling or drought imposition. This ensured that drought stress was strictly regulated based on the evaporation pan measurements.

The irrigation volume required for each treatment was calculated based on these specific levels of evaporation, and was subsequently applied to the respective plots using a volumetric meter. To prevent water penetration and the potential mixing of treatment effects, the ends of the plots were completely sealed. Irrigation was conducted independently for each plot and block using a nozzle and volume meter. Throughout the growing season, the implementation of necessary pest control measures, weeding, and meticulous record-keeping was performed to maintain the overall health and integrity of the experimental plots.

During the growth period, the greenness of 10 fully developed and mature leaves was measured using a chlorophyll meter (Minolta SPAD unit 502). Concurrently, the leaf area of selected plants (excluding marginal effects) was determined using a leaf area measuring device (Delta T Devices, Cambridge, UK). Upon reaching full ripening, a random selection was made of half a metre from each experimental plot, accounting for any marginal effects. Subsequent measurements were taken of various traits, including the dry weight of aerial parts (leaves and stems), the number of branches, the number of capsules per plant, the number of seeds in each capsule, the weight of 1000 seeds, seed yield, and biological yield. The harvest index was calculated using equation (1):

(1) HI=(seed Yield/ Biological Yield) × 100

To determine the percentage of oil, 10 gr of powdered samples were subjected to extraction using a Soxhlet apparatus and petroleum ether solvent. A rotary device was employed to remove the solvent from the samples. The oil percentage of each sample was calculated by weighing the extracted oil. The oil and meal yield were obtained by multiplying the oil and meal percentages with the grain yield, respectively.

The water use efficiency (WUE) was assessed by measuring the relative humidity of the soil and determining the volume of water utilised at each stage prior to irrigation. In the experimental setup, different irrigation volumes were applied for normal and drought stress conditions. Under normal conditions, an irrigation volume of 4100 m^3^/ha was applied, while for drought stress conditions, the irrigation volume was reduced to 3100 m^3^/ha.

The WUE was calculated using the following equation (2) [18]:

(2) WUE = D/W

Where:

WUE represents the water use efficiency, measured in kg of product per m^3^ of water.

D corresponds to the mass of dry matter of the seed produced, measured in kg.

W denotes the volume of water used, measured in m^3^.

### 2.1 Meta-analysis for estimating % change

Individual effect sizes were computed as log ratios (lnR) in equation (3) for each response variable. Each value of lnR represents the natural logarithm of the ratio of the mean value of a treatment group (fertilizer application treatments; ${\bar{x}}_{t}$) to the mean value of the corresponding control group (control treatment; ${\bar{x}}_{c}$):

(3) $$lnR=\ln\;\frac{{\bar{x}}_{t}}{{\bar{x}}_{c}}$$

The *lnR* is a widely adopted metric in meta-analyses for expressing treatment effects on a consistent scale in agriculture science [20]. Values of *lnR* were derived for each response variable (Leaf area index, Number of branches, number of capsules, number of seeds per capsules, thousand seed weight, green yield, Biological yield, Harvest index, Oil yield, Meal yield and Water use efficiency) and each study locaation.

For fertilizer application treatments and for each response variable, the mean value of *lnR* and its 95 % confidence intervals (CI) was estimated with the *rma()* function of the metafor package [21] using the R software (version 4.2.3). Following Borenstein et al. (2021), we defined a hierarchical statistical model including a random study effect as follows: ${lnR}_{i}=\mu +b_{i}+e_{i}$, with $b_{i}∼N\left(0,\sigma_{b}^{2}\right)$ and $e_{i}∼N\left(0,\sigma_{i}^{2}\right)$, where ${lnR}_{i}$ was the log ratio in study *i*, *i* = 1, …, *n*, μ was the mean effect size, $\sigma_{b}^{2}$ and $\sigma_{i}^{2}$ were the between-tretments and within-treatments variances, respectively, *n* was the total number of replication. The values of $\sigma_{i}^{2}$, *i* = 1, …, *n*, were estimated from the sample sizes extracted from the *n* selected treatments. The variance $\sigma_{b}^{2}-$ describing the heterogeneity of ${lnR}_{i}$ across treatments – was estimated by Restricted Maximum Likelihood with the *rma* function. Then, the mean value of *lnR* (i.e., μ) was estimated with *rma* as the weighted sum of ${lnR}_{i}$, using weights expressed as $\frac{1}{\sigma_{b}^{2}+\sigma_{i}^{2}}$. The heterogeneity of the effect sizes (i.e., the variability of ${lnR}_{i}$ across studies) was tested using a Q statistical test [22] with *rma*. This approach was implemented to each response variable and to fertilizer application, In each location separately.

To facilitate the understanding and interpretation of the results, we expressed the mean effect size (and its 95 % confidence interval) as a percentage of variation of the response attributable to fertilizer application compared to normal control treatment.

The relationship of traits to effect size (defined as log risk ratio) was calculated as equation (4):

(4) Ln (RR) = intercept – the slope of each covariate(X)

where X is the absolute value of traits.

The 95 % confidence interval for each covariate was estimated as equations (5), (6)):

(5) LL=X−1.96×SE

(6) UL=X+1.96×SE

with LL and UL referring to lower and upper limits, respectively. In the above-mentioned equations, 1.96 shows the Z-value corresponding to confidence limits of 95 %.

The data analysis was performed using SAS 9.4 software. Maps illustrating the results were created using R software and Excel software. As the experiment was conducted at two separate locations, Bartlett's test was performed prior to the analysis of variance (ANOVA) to ensure the homogeneity of variances across datasets.Following this, the results and non-significant of the variance of locations in Bartlett analysis, a two-way ANOVA was applied to evaluate the main effects of location, treatments, and their interactions.The Duncan multiple-range test was utilised to evaluate the variation among the treatment means.

## 3 Results

The results of this study showed that the main effects of location, irrigation, fertilizer, and their interactions had significant effects on leaf area index (LAI), number of branches, number of capsules, number of seeds per capsule, and seed yield. However, there was no significant effect on thousand seed weight. This means that all of these traits were affected by the experimental factors, as shown in Table 2.

**Table 2:** Results of analysis of variance of yield and yield components of sesame under different fertilizer application and drought stress in two locations, Dashthouz and Sarkahnan.

S.O.V	Df	LAI	Number of branches	Number of Capsules	Number of Seeds per Capsule	Thousand seed weight	seed yield
Location	1	1.26∗	0.10ns	29751.04∗∗	5711.87∗∗	0.0009ns	88865.34∗∗
Rep (Location)	4	0.11ns	0.21ns	120.23	32.52	0.08	844.59
Irrigation	1	53.40∗∗	3.56∗∗	794.65∗	633.96∗∗	0.78ns	77736.78∗∗
Fertilizers	7	10.49∗∗	17.58∗∗	4658.23∗∗	213.75∗∗	0.01ns	37164.19∗∗
Location ∗ Irrigation	1	15.20∗∗	1.10∗	1539.20∗∗	318.64∗∗	0.01ns	22022.04∗∗
Location ∗ Fertilizers	7	0.82∗∗	0.67∗	437.33∗∗	45.82∗	0.04ns	1095.91ns
Irrigation ∗ Fertilizers	7	0.72∗∗	1.34∗∗	201.08ns	70.45∗∗	0.01ns	4728.96∗∗
Location ∗ Irrigation ∗ Fertilizers	7	0.62∗	1.57∗	320.84∗	56.01∗∗	0.02ns	2631.78∗
Error	60	0.21	0.29	147.81	18.94	0.02	1247.36
CV (%)	–	14.61	15.55	20.27	10.26	4.61	16.89

∗, ∗∗ and NS are respectively significant at 5 and 1 % probability level and non-significant.

The results presented in Table 3 demonstrate significant variations in the measured agronomic traits across different fertilizer treatments, irrigation conditions, and locations. For leaf area index (LAI), the lowest value of 1.4 was observed in the control treatment under drought stress conditions, while the highest value of 5.5 was obtained in the B+NPK under normal irrigation conditions. These findings indicate the sensitivity of LAI to environmental conditions and the potential influence of fertilization on leaf development. Regarding the number of branches, the lowest value of 3.16 was obtained in the control treatment under drought stress in Dashthouz, whereas the highest value of 10.16 was observed in the B+MY+NPK treatment under normal irrigation in the same location. These contrasting results suggest that different fertilizer combinations and irrigation conditions can significantly affect branching patterns and plant architecture. In terms of the number of capsules, the lowest value of 20.6 was recorded in the control treatment under drought stress in Sarkahnan, while the highest value of 115.7 was observed in the B+MY+NPK treatment under normal irrigation in the same location. These findings indicate the influence of fertilizer treatments and irrigation on reproductive structures and potential seed production. For the number of seeds per capsule, the lowest value of 22.7 was observed in the control treatment under both normal irrigation conditions in Dashthouz and Sarkahnan. In contrast, the highest value of 55.7 was obtained in the B+MY+NPK treatment under normal irrigation in Sarkahnan. These results highlight the potential for enhanced seed production through specific fertilizer combinations and optimal irrigation practices. Lastly, in terms of seed yield, the lowest value of 95.3 gr/m^2^ was observed in the control treatment under normal irrigation conditions in Dashthouz, while the highest value of 325.5 gr/m^2^ was obtained in the control treatment under normal irrigation conditions in Sarkahnan. These findings emphasise the significance of location-specific factors and their interaction with irrigation and fertilizer treatments in determining overall seed yield. In summary, the findings of this study provide valuable insights into the effects of different fertilizer treatments and irrigation conditions on various agronomic traits. The observed variations in LAI, number of branches, number of capsules, number of seeds per capsule, and seed yield underscore the importance of tailored fertilization and irrigation strategies for optimizing plant growth and productivity.

**Table 3:** Mean comparison of yield and yield components of sesame under location, irrigation and fertilizer application treatments.

Location	Irrigation	Fertilizers	LAI	Number of branches	Number of Capsules	Number of Seeds per Capsule	seed yield (gr/m^2^)
Dashthouz	Normal	B+NPK	5.5a	8.86a-e	73.4def	49.4bcd	292.8a-d
		B+MY	2.4f-i	5.58hij	47.3hij	54.2 ab	231.2e-i
		B+MY+NPK	4.9a	10.16a	62.1fgh	55.6 ab	310.2abc
		MY+NPK	3.8bc	9.31abc	62.5fgh	54.3 ab	266.8b-f
		B	1.9g-l	4.15j-m	56.5fgh	37.6 fg	142.5k-n
		Control	1.9g-l	3.53m	26.5 kl	32.7gh	124.8mn
		NPK	3.9bc	9.48abc	69.6efg	55.1 ab	288.4a-e
		MY	2.6efg	5.51h-k	30.6jkl	38.6efg	191.1h-k
	Drought Stress	B+NPK	2.9def	8.37b-f	34.6i-l	45.1cde	212.1f-j
		B+MY	2.16g-j	3.9klm	26.4 kl	46.2cd	201.3g-j
		B+MY+NPK	4.1b	8.37b-f	47.2hij	34.4gh	232.1f-j
		MY+NPK	3.8bc	9.17a-d	49.2hij	38.1efg	201.3g-j
		B	2.1g-k	4.71i-m	47.5hij	33.5gh	104.2n
		Control	1.4 kl	3.16m	22.7l	28.4h	95.3n
		NPK	3.4bcd	8.12c-f	47.3hij	43.3def	212.1f-j
		MY	1.5jkl	4.15j-m	22.7l	33.5gh	175.3i-m
Sarkahnan	Normal	B+NPK	4.9a	9.12a-e	100.9abc	55.7 ab	325.5a
		B+MY	4.1b	4.71i-m	62.3fgh	42.7def	236.3d-h
		B+MY+NPK	5.3a	9.85 ab	115.7a	45.1cde	296.8abc
		MY+NPK	4.8a	8.61a-e	94.4bc	53.7 ab	289.4a-d
		B	3.5bcd	5.71hij	57.2fgh	30.4h	191.8h-k
		Control	3.2cde	5.39h-l	34.8i-l	30.4h	121.5mn
		NPK	5.5a	9.21a-e	108.4 ab	57.6a	316.5 ab
		MY	3.9bc	4.43i-m	61.5fgh	37.7 fg	191.4h-k
	Drought Stress	B+NPK	2.4fgh	6.94fgh	84.3cde	53.7 ab	259.3b-f
		B+MY	1.7h-l	4.27j-m	53.5ghi	42.9def	171.3j-m
		B+MY+NPK	3.3bcde	7.44efg	110 ab	38efg	310.6abc
		MY+NPK	1.9g-l	7.62def	92.7bcd	51.5abc	253.1c-g
		B	1.6i-l	4.21j-m	45.4h-k	29.4h	172.6j-m
		Control	1.3l	3.84lm	20.6l	27.7h	102.5n
		NPK	2.9def	8.96a-e	94bc	53.6 ab	267.9a-f
		MY	1.6jkl	4.42i-m	55.3fgh	31.8gh	183.4h-l

In each column and for each treatment, means marked with the same alphabetical letters are not significantly different, based on Duncan's multiple range test at the 5 % significance level.

The study demonstrated considerable impacts of various factors, including location, irrigation, and fertilizers, on multiple aspects of sesame cultivation. Notably, biological yield, harvest index, oil yield, meal yield, and water use efficiency were all significantly affected by these main factors and their interactions. The results underscore the significance of tailoring agricultural practices to suit specific location-specific conditions, optimizing irrigation techniques, and adopting suitable fertilizer strategies. These measures are essential for improving the overall productivity and resource utilization of sesame crops, as revealed by the detailed findings presented in Table 4.

**Table 4:** Results of analysis of variance of biological yield, harvest index (HI), Oil yield, Meal yield, and water use efficiency (WUE) of sesame under different treatments, including location, irrigation, and fertilizer application.

S.O.V	Df	Biological Yield	HI	Oil yield	Meal Yield	WUE
Location	1	5820512.78∗∗	214.20∗	20959.81∗∗	18785.21∗∗	44.55∗∗
Rep (Location)	4	31731.91	12.26	133.61	165.96	0.29
Irrigation	1	412165.35∗∗	300.33∗∗	9474.41∗∗	22134.26∗∗	5.65∗∗
Fertilizers	7	1090678.07∗∗	17.32ns	6472.85∗∗	10553.09∗∗	8.80∗∗
Location ∗ Irrigation	1	161187.45∗∗	479.72∗∗	4940.70∗∗	13687.53∗∗	0.03
Location ∗ Fertilizers	7	200537.39∗∗	58.55∗∗	564.54∗∗	608.62∗∗	1.48∗∗
Irrigation ∗ Fertilizers	7	35283.97∗	45.33∗	842.90∗∗	2680.20∗∗	0.51∗∗
Location ∗ Irrigation ∗ Fertilizers	7	62517.00∗∗	53.97∗∗	300.54∗∗	614.27∗∗	0.40∗∗
Error	60	15798.01	16.07	94.93	203.73	0.13
CV (%)	–	11.54	20.12	10.72	12.44	12.09

∗ and ∗∗ are respectively significant at 5 and 1 % probability level.

Table 5 presents the results of the study, showcasing significant variations in agronomic traits such as biological yield, harvest index (HI), oil yield, meal yield, and water use efficiency (WUE) across different locations, irrigation conditions, and fertilizer treatments. The data allows us to gain valuable insights into the performance range exhibited within the dataset.

**Table 5:** Mean comparison of biological yield, harvest index, oil and meal yield and water use efficiency of sesame under location, irrigation and fertilizer application treatments.

Location	Irrigation	Fertilizers	Biological Yield (gr/m^2^)	HI (%)	Oil yield (gr/m^2^)	Meal Yield (gr/m^2^)	WUE (kg/m^3^)
Dashthouz	Normal	B+NPK	1267.1d	23.2c-f	132.6abc	193.5a	3.39ghi
		B+MY	934.1f-i	25.3bcd	87.66j-m	133.5ef	2.28j-m
		B+MY+NPK	1106.1def	33.1a	108.9f-h	164.6bc	2.37k-o
		MY+NPK	1095.3def	26.9bc	103.4g-j	133.4ef	2.4j-m
		B	744.23i-l	21.8c-g	76.67lm	98.83g	1.81m-q
		Control	580.8klm	19.7d-i	55.83op	69.54jkl	1.52opq
		NPK	1169.5de	30.5b	127.4bcd	161.4bcd	2.31j-n
		MY	918.3f-i	24b-e	79.93klm	92.13g-j	1.41pq
	Drought Stress	B+NPK	953.4fgh	23.1b-e	74.6lm	163.5abc	3.28ghi
		B+MY	842.3ghi	24.7b-e	76.6lm	110.5 fg	1.75n-q
		B+MY+NPK	1005.4efg	26.4bcd	82.2klm	127.8ef	3.18f-i
		MY+NPK	986.6efg	18.4e-i	90.7i-l	110.5 fg	3.53efg
		B	621.3j-m	20.4d-i	52.9op	91.56gh	2.32l-o
		Control	425.8m	16.7f-i	45.6op	59.74l	2.42j-m
		NPK	948.1f-i	23.1b-e	120c-f	107.8g	3.77def
		MY	805.3g-j	21.2c-h	87.9j-m	96.63gh	1.37q
Sarkahnan	Normal	B+NPK	1711.4b	19.1d-i	141.5 ab	183.9 ab	4.17cd
		B+MY	1085def	19.3d-i	94.63h-k	111.3 fg	2.64h-k
		B+MY+NPK	2024.6a	24.1b-e	145.8a	164.7bc	4.93 ab
		MY+NPK	1619.7bc	17.9e-i	112d-g	143.9cde	3.95de
		B	1098.1def	19.7d-i	82.26klm	95.06ghi	2.68hij
		Control	749.7h-k	18.1e-i	52.96op	68.56 kl	2.24j-n
		NPK	1967.4a	17.4f-i	144.8a	171.7 ab	4.79b
		MY	985.5efg	17.3f-i	81.43klm	133.4ef	2.69hij
	Drought Stress	B+NPK	1571.4bc	16.6f-i	120.7c-f	138.5de	5.07 ab
		B+MY	835.3ghi	15.1hi	73.4mn	94.9ghi	2.69hij
		B+MY+NPK	1715.5b	19.2d-i	135.4abc	160.9bcd	5.53a
		MY+NPK	1480.8c	17.1f-i	106.5f-i	140.2de	4.78bc
		B	872.2ghi	17.6e-i	76.7lm	90.4g-k	2.81hij
		Control	623.9j-m	13.6i	48.5op	57.33l	2.6i-l
		NPK	1541.4bc	16.4ghi	123.5cde	144.6cde	4.97 ab
		MY	850.5ghi	17.9e-i	83.2klm	96.2ghi	3.25fgh

In each column and for each treatment, means marked with the same alphabetical letters are not significantly different, based on Duncan's multiple range test at the 5 % significance level.

For the biological yield trait, the lowest recorded value was 425.8 gr/m^2^, observed in the Dashthouz location under drought stress conditions, specifically in the control treatment. On the other end of the spectrum, the highest value of 2024.6 gr/m^2^ was recorded in the Sarkahnan location under normal irrigation conditions, particularly in the B+MY+NPK treatment. These findings underscore the considerable influence of location-specific factors and the use of specific fertilizer treatments on the overall biomass production of sesame crops. Such variations in biological yield highlight the importance of optimizing irrigation and fertilizer practices to achieve higher crop productivity in diverse environmental conditions.Regarding the harvest index, the lowest value of 13.6 % was observed in the control treatment under normal irrigation conditions in Sarkahnan, whereas the highest value of 33.1 % was recorded in the Dashthouz location under normal irrigation conditions, specifically in the B+MY+NPK treatment. These results indicate differences in the proportion of harvested product relative to the total biological yield, implying variations in resource allocation efficiency among the treatments.

For oil yield, the lowest value of 45.6 gr/m^2^ was observed in the control treatment under normal irrigation conditions in Sarkahnan, while the highest value of 145.8 gr/m^2^ was recorded in the B+MY+NPK treatment in the same location. These results highlight the potential influence of fertilizer treatments on the oil production potential of the crops. In terms of meal yield, the lowest value of 57.33 gr/m^2^ was observed in the control treatment under normal irrigation conditions in Sarkahnan, while the highest value of 193.5 gr/m2 was recorded in the B+NPK treatment in Dashthouz. These findings indicate differences in the amount of residual material obtained after oil extraction, which may have implications for further utilization of the byproduct. Regarding water use efficiency (WUE), the lowest value of 1.37 kg/m^3^ was observed in the MY treatment in Dashthouz under normal irrigation conditions, while the highest value of 5.53 kg/m^3^was recorded in the B+MY+NPK treatment in the same location under normal irrigation conditions. These results demonstrate variations in the efficiency of water utilization by the crops under different fertilizer treatments and highlight the potential for optimizing water management practices. In summary, the presented findings provide valuable insights into the performance of agronomic traits in relation to location, irrigation conditions, and fertilizer treatments. The observed variations in biological yield, harvest index, oil yield, meal yield, and water use efficiency highlight the importance of optimizing fertilizer strategies, irrigation practices, and crop management techniques to enhance crop productivity, resource utilization, and sustainability. Further research is warranted to fully understand the underlying mechanisms and interactions influencing these traits and to develop targeted strategies for improving crop performance and resource use efficiency.

### 3.1 Evolution effects size and estimate percentage changes of traits

#### 3.1.1 LAI

**Fig. 2:**
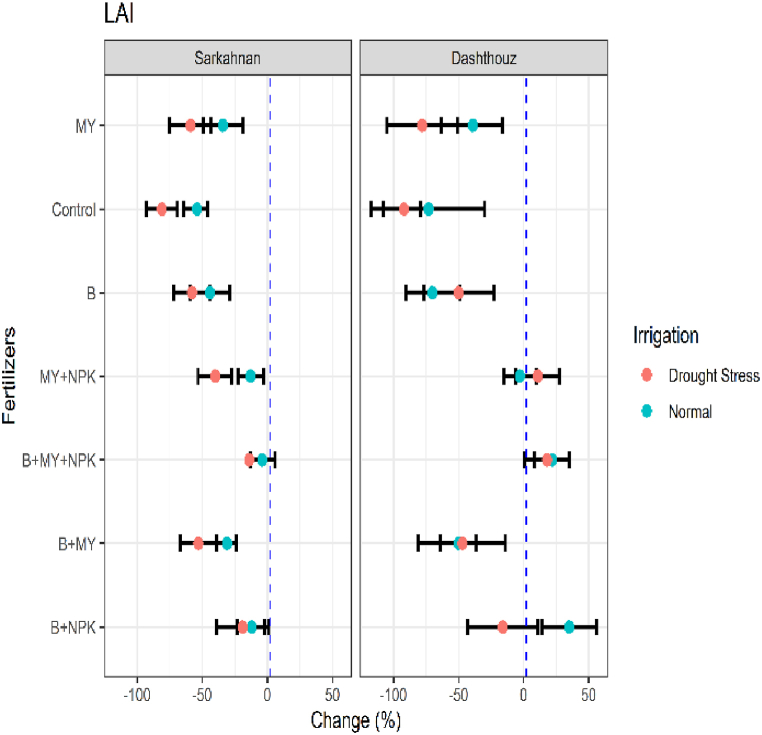
Percentage changes in leaf area index (LAI) in two locations (Dashthouz and Sarkahnan) under drought stress and normal conditions with the application of different types of fertilizers. (the changes are calculated compared to the chemical fertilizer (NPK) treatment).

In Sarkahnan, under normal irrigation, the lowest percentage change in leaf area index (LAI) was observed in the B treatment (−44 %), while the highest percentage change was found in the B+NPK treatment (12 %). Under drought stress in Sarkahnan, the lowest percentage change was observed in the control treatment (−81 %), while the highest percentage change was seen in the B+MY treatment (−53 %). In Dashthouz, under normal irrigation, the lowest percentage change was observed in the B treatment (−70 %), while the highest percentage change was found in the B+NPK treatment (35 %). Under drought stress in Dashthouz, the lowest percentage change was observed in the control treatment (−92 %), and the highest percentage change was seen in the B+MY+NPK treatment (18 %) (Fig. 2). These results highlight the varying effects of different fertilizer treatments and irrigation conditions on the leaf area index of sesame plants.

#### 3.1.2 Number of branches

**Fig. 3:**
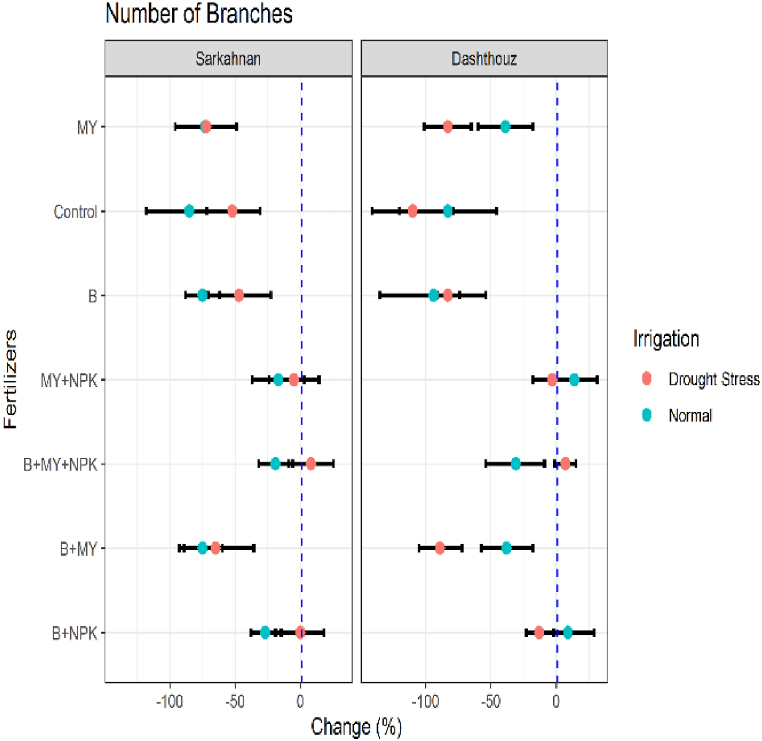
Percentage changes in number of branches in two locations (Dashthouz and Sarkahnan) under drought stress and normal conditions with the application of different types of fertilizers. (the changes are calculated compared to the chemical fertilizer (NPK) treatment).

Based on the presented results of Fig. 3 the mean percentage changes in the number of branches for different locations, fertilizer treatments, and irrigation conditions. In Sarkahnan under normal irrigation, the B+NPK treatment showed a decrease in the number of branches by 27 %, while the B+MY treatment exhibited a larger decrease of 75 %. The B+MY+NPK treatment showed a smaller decrease of 19 %, and the MY+NPK treatment displayed a decrease of 17 %. The B, control, and MY treatments resulted in significant decreases in the number of branches by 75 %, 85 %, and 73 %, respectively. Under drought stress in Sarkahnan, the B+NPK treatment showed no significant change in the number of branches, indicating comparable growth to the reference treatment. However, the B+MY treatment exhibited a larger decrease of 65 %. The B+MY+NPK treatment showed a small increase of 8 %, and the MY+NPK treatment exhibited a decrease of 5 %. The B, control, and MY treatments resulted in significant decreases in the number of branches by 47 %, 52 %, and 72 %, respectively. In Dashthouz under normal irrigation, the B+NPK treatment showed an increase in the number of branches by 9 %, indicating improved growth compared to the reference treatment. However, the B+MY treatment displayed a decrease of 38 % in the number of branches, while the B+MY+NPK treatment showed a decrease of 31 %. The MY+NPK treatment exhibited an increase of 14 %, and the B, control, and MY treatments resulted in significant decreases in the number of branches by 94 %, 83 %, and 39 %, respectively. Under drought stress in Dashthouz, the B+NPK treatment showed a decrease in the number of branches by 13 %, while the B+MY treatment exhibited a larger decrease of 89 %. The B+MY+NPK treatment showed a small increase of 7 %, and the MY+NPK treatment exhibited a decrease of 3 %. The B, control, and MY treatments resulted in significant decreases in the number of branches by 83 %, 110 %, and 83 %, respectively.

#### 3.1.3 Number of capsules

**Fig. 4:**
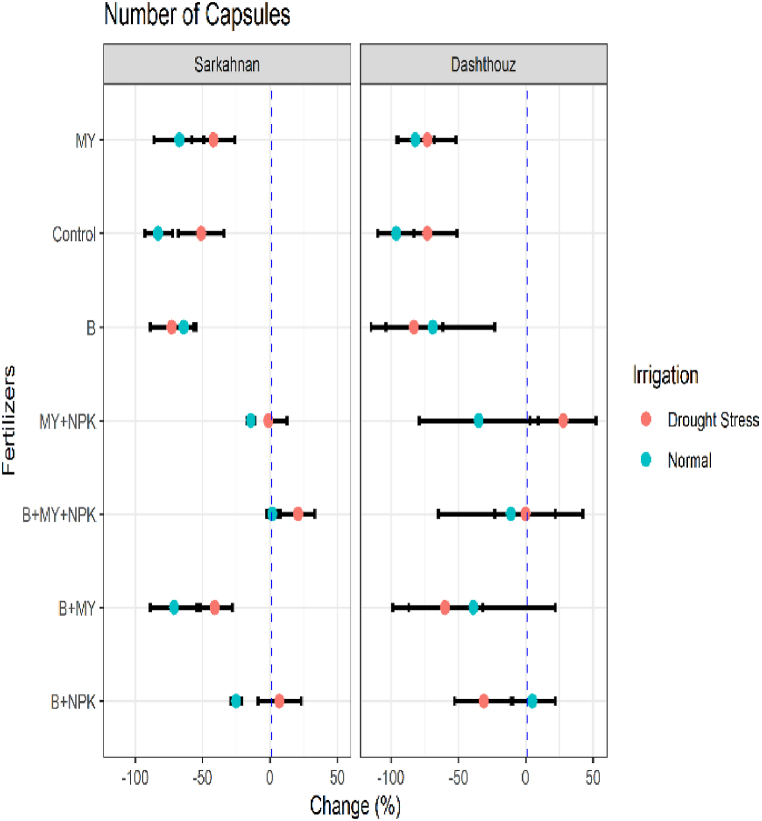
Percentage changes in number of capsules in two locations (Dashthouz and Sarkahnan) under drought stress and normal conditions with the application of different types of fertilizers. (the changes are calculated compared to the chemical fertilizer (NPK) treatment).

Fig. 4 presents the mean percentage changes in the number of capsules for different locations, fertilizer treatments, and irrigation conditions. In Sarkahnan under normal irrigation, the B+NPK treatment showed a decrease in the number of capsules by 25 %, while the B+MY treatment exhibited a larger decrease of 71 %. The B+MY+NPK treatment showed a slight increase of 2 %, and the MY+NPK treatment displayed a decrease of 14 %. The B, control, and MY treatments resulted in significant decreases in the number of capsules by 64 %, 83 %, and 67 %, respectively. Under drought stress in Sarkahnan, the B+NPK treatment showed an increase in the number of capsules by 7 %, indicating improved performance compared to the reference treatment. However, the B+MY treatment exhibited a decrease of 41 % in the number of capsules, while the B+MY+NPK treatment showed a small increase of 21 %. The MY+NPK treatment exhibited a slight decrease of 1 %. The B, control, and MY treatments resulted in significant decreases in the number of capsules by 73 %, 51 %, and 42 %, respectively. In Dashthouz under normal irrigation, the B+NPK treatment showed a slight increase in the number of capsules by 5 %, indicating enhanced performance compared to the reference treatment. However, the B+MY treatment displayed a decrease of 39 % in the number of capsules, while the B+MY+NPK treatment showed a decrease of 11 %. The MY+NPK treatment exhibited a decrease of 35 %, and the B, control, and MY treatments resulted in significant decreases in the number of capsules by 69 %, 96 %, and 82 %, respectively. Under drought stress in Dashthouz, the B+NPK treatment showed a decrease in the number of capsules by 31 %, while the B+MY treatment exhibited a larger decrease of 60 %. The B+MY+NPK treatment showed a minimal change of −0.1 %, and the MY+NPK treatment exhibited an increase of 28 %. The B, control, and MY treatments resulted in significant decreases in the number of capsules by 83 %, 73 %, and 73 %, respectively.

#### 3.1.4 Number of seed per capsules

**Fig. 5:**
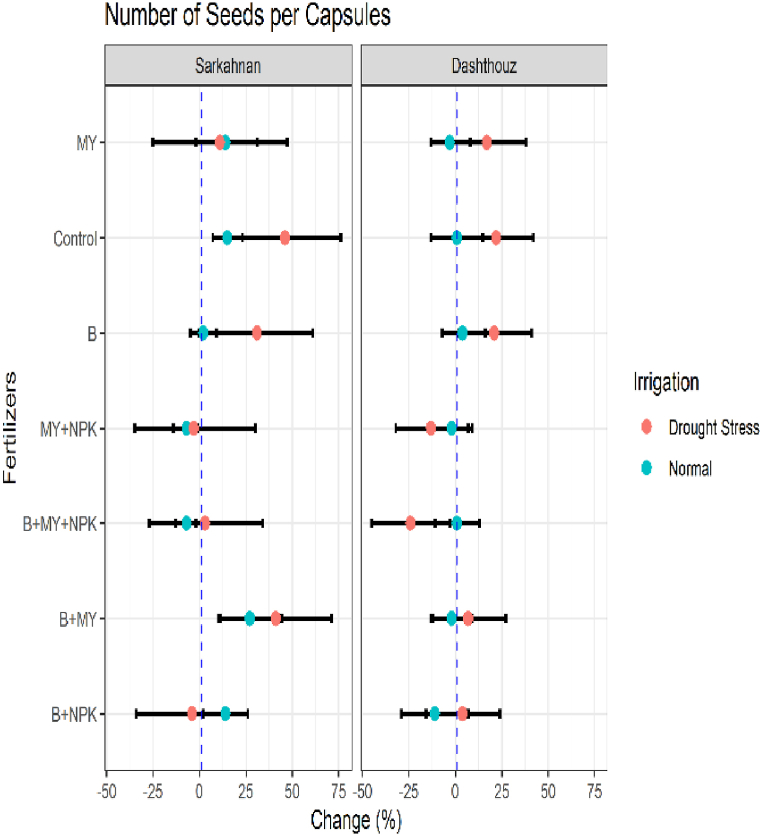
Percentage changes in number of seeds per capsules in two locations (Dashthouz and Sarkahnan) under drought stress and normal conditions with the application of different types of fertilizers. (the changes are calculated compared to the chemical fertilizer (NPK) treatment).

The results indicated that the mean number of seeds per capsule for different locations, fertilizer treatments, and irrigation conditions. In Sarkahnan under normal irrigation, the B+NPK treatment exhibited an increase of 14 % in the number of seeds per capsule, while the B+MY treatment showed a higher increase of 27 %. The B+MY+NPK and MY+NPK treatments displayed slight decreases of −7% and −7%, respectively. The B and control treatments resulted in a small increase of 2 % and 15 %, respectively, in the number of seeds per capsule. Under drought stress in Sarkahnan, the B+NPK treatment showed a decrease of −4% in the number of seeds per capsule, while the B+MY treatment exhibited a significant increase of 41 %. The B+MY+NPK treatment displayed a slight increase of 3 %, and the MY+NPK treatment exhibited a decrease of −3%. The B treatment resulted in a significant increase of 31 % in the number of seeds per capsule, while the control treatment showed an even higher increase of 46 %. The MY treatment displayed a modest increase of 11 %. In Dashthouz under normal irrigation, the B+NPK treatment showed a slight decrease of −11 % in the number of seeds per capsule, while the B+MY treatment exhibited a minimal change of −2%. The B+MY+NPK and MY+NPK treatments displayed slight increases of 1 % and −2%, respectively. The B treatment resulted in a small increase of 4 %, while the control treatment and MY treatment showed minimal changes of 1 % and −3%, respectively, in the number of seeds per capsule. Under drought stress in Dashthouz, the B+NPK treatment exhibited an increase of 4 % in the number of seeds per capsule, while the B+MY treatment showed a slightly higher increase of 7 %. The B+MY+NPK treatment displayed a significant decrease of −24 %, and the MY+NPK treatment exhibited a decrease of −13 %. The B treatment resulted in a notable increase of 21 % in the number of seeds per capsule, while the control treatment showed a slightly higher increase of 22 %. The MY treatment displayed an increase of 17 % (Fig. 5).

#### 3.1.5 Thousand seed weight

**Fig. 6:**
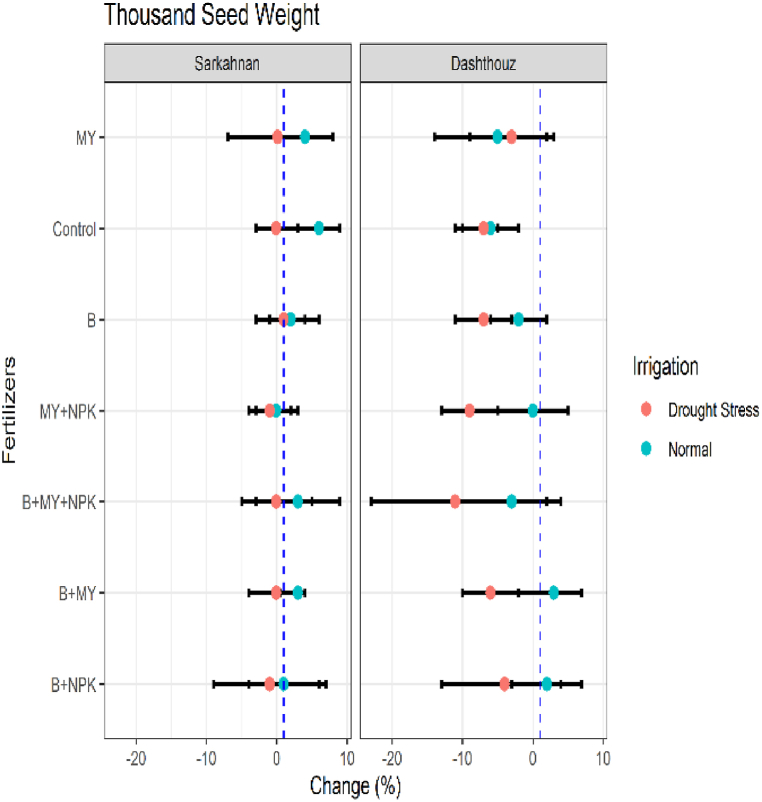
Percentage changes in thousand seed weight in two locations (Dashthouz and Sarkahnan) under drought stress and normal conditions with the application of different types of fertilizers. (the changes are calculated compared to the chemical fertilizer (NPK) treatment).

In Sarkahnan under normal irrigation, the lowest percentage change in the thousand seed weight was observed in the MY+NPK treatment (−0.1 %), while the highest percentage change was found in the control treatment (6 %). Under drought stress in Sarkahnan, the lowest percentage change was observed in the B+NPK treatment (−1%), and the highest percentage change was seen in the B treatment (1 %). In Dashthouz under normal irrigation, the lowest percentage change was observed in the B+MY+NPK treatment (−3%), while the highest percentage change was found in the B+MY treatment (3 %). Under drought stress in Dashthouz, the lowest percentage change was observed in the B+MY+NPK treatment (−11 %), and the highest percentage change was seen in the B+MY treatment (−6%) (Fig. 6).

#### 3.1.6 Sees yield

**Fig. 7:**
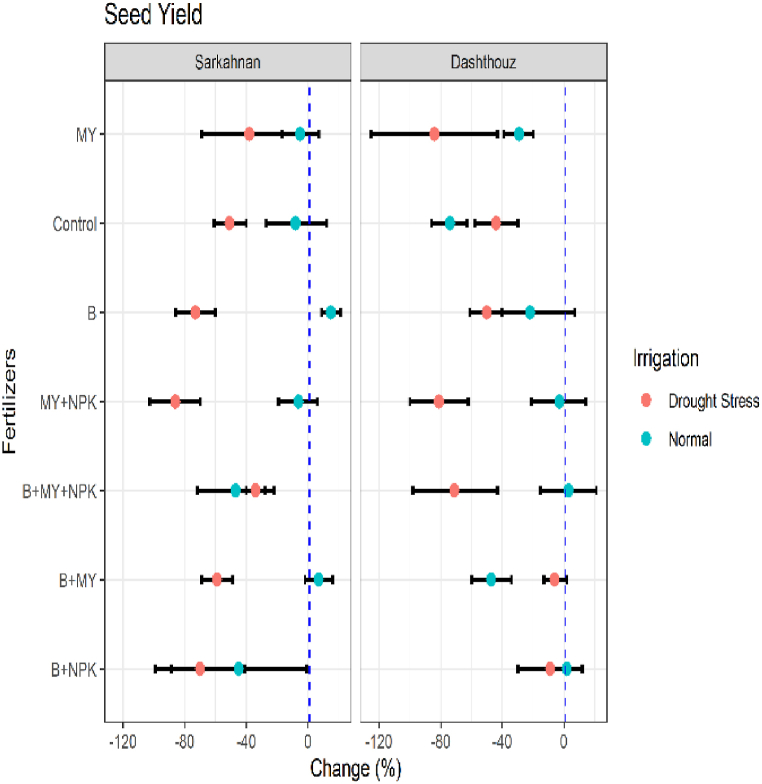
Percentage changes in seed yield in two locations (Dashthouz and Sarkahnan) under drought stress and normal conditions with the application of different types of fertilizers. (the changes are calculated compared to the chemical fertilizer (NPK) treatment).

The results depicted in Fig. 7 provide insights into the percentage changes in the seed yield of sesame compared to the NPK fertilizer treatment. The findings demonstrate notable variations among different treatments in both Sarkahnan and Dashthouz locations under normal irrigation and drought stress conditions. In Sarkahnan under normal irrigation, the B+NPK treatment exhibited the highest decrease in seed yield, with a percentage change of −45 %. Conversely, the B treatment showed a modest increase of 15 % compared to the NPK fertilizer treatment. Under drought stress in Sarkahnan, the MY+NPK treatment had the most substantial decrease in seed yield, with a percentage change of −86 %. Similarly, the B treatment displayed a considerable decrease of −73 %. In contrast, the B+MY treatment showed a relatively smaller decrease of −59 %. Moving to Dashthouz under normal irrigation, the B+MY+NPK treatment demonstrated the highest increase in seed yield, with a percentage change of 3 %. On the other hand, the B+MY treatment exhibited the highest decrease, with a percentage change of −47 %. Under drought stress in Dashthouz, the MY treatment had the most significant decrease in seed yield, with a percentage change of −84 %. Likewise, the B+MY+NPK treatment displayed a substantial decrease of −71 %. Meanwhile, the B+NPK treatment showed a relatively smaller decrease of −9%.

#### 3.1.7 Biological yield

**Fig. 8:**
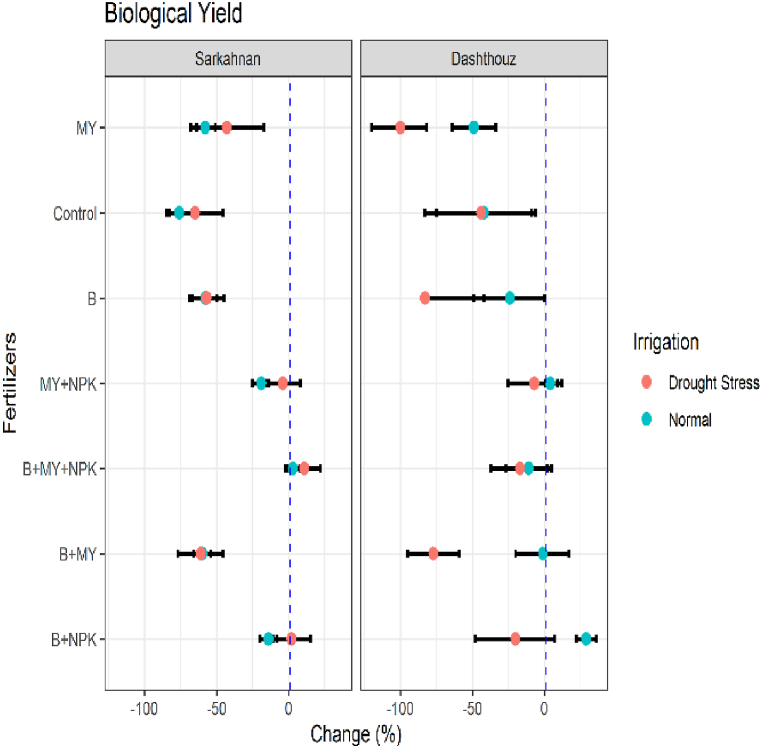
Percentage changes in biological yield in two locations (Dashthouz and Sarkahnan) under drought stress and normal conditions with the application of different types of fertilizers. (the changes are calculated compared to the chemical fertilizer (NPK) treatment).

Fig. 8 illustrates the percentage changes in the biological yield of sesame compared to the NPK fertilizer treatment. The results reveal significant variations among different treatments in both Sarkahnan and Dashthouz locations under normal irrigation and drought stress conditions. In Sarkahnan under normal irrigation, the B+MY treatment displayed the highest decrease in the biological yield, with a percentage change of −60 %. This was followed by the B treatment, which showed a decrease of −58 %. Conversely, the B+NPK treatment exhibited a relatively smaller decrease of −14 %. Under drought stress in Sarkahnan, the B+MY treatment had the highest decrease in the biological yield, with a percentage change of −61 %. The B treatment also showed a substantial decrease of −57 %. On the other hand, the B+MY+NPK treatment exhibited a moderate increase of 11 %. Moving to Dashthouz under normal irrigation, the B+NPK treatment exhibited the highest increase in the biological yield, with a percentage change of 29 %. In contrast, the B+MY treatment showed a slight decrease of −1%. Under drought stress in Dashthouz, the B treatment had the highest decrease in the biological yield, with a percentage change of −83 %. The B+MY treatment also displayed a significant decrease of −77 %. Meanwhile, the B+MY+NPK treatment showed a moderate decrease of −17 %.

#### 3.1.8 Harvest index

**Fig. 9:**
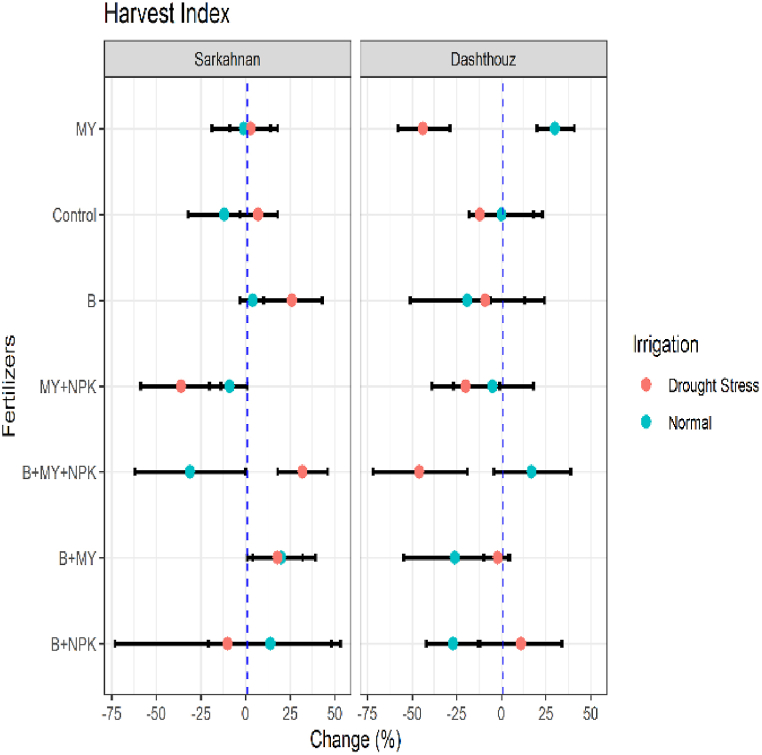
Percentage changes in harvest index in two locations (Dashthouz and Sarkahnan) under drought stress and normal conditions with the application of different types of fertilizers. (the changes are calculated compared to the chemical fertilizer (NPK) treatment).

In Sarkahnan under normal irrigation, the B+MY treatment had the highest increase in the harvest index, with a percentage change of 20 %. This was followed by the B+NPK treatment, which showed an increase of 14 %. On the other hand, the B+MY+NPK treatment exhibited the highest decrease in the harvest index, with a percentage change of −31 %. Under drought stress in Sarkahnan, the B+MY+NPK treatment displayed the highest increase in the harvest index, with a percentage change of 32 %. The B+MY treatment also showed a significant increase of 18 %. Conversely, the MY+NPK treatment had the highest decrease in the harvest index, with a percentage change of −36 %. in Dashthouz location under normal irrigation, the B+MY+NPK treatment exhibited the highest increase in the harvest index, with a percentage change of 17 %. Meanwhile, the B+MY treatment showed the highest decrease, with a percentage change of −26 %. Under drought stress in Dashthouz, the B+MY+NPK treatment had the highest decrease in the harvest index, with a percentage change of −46 %. On the other hand, the MY treatment showed the highest increase, with a percentage change of 11 % (Fig. 9).

#### 3.1.9 Oil yield

**Fig. 10:**
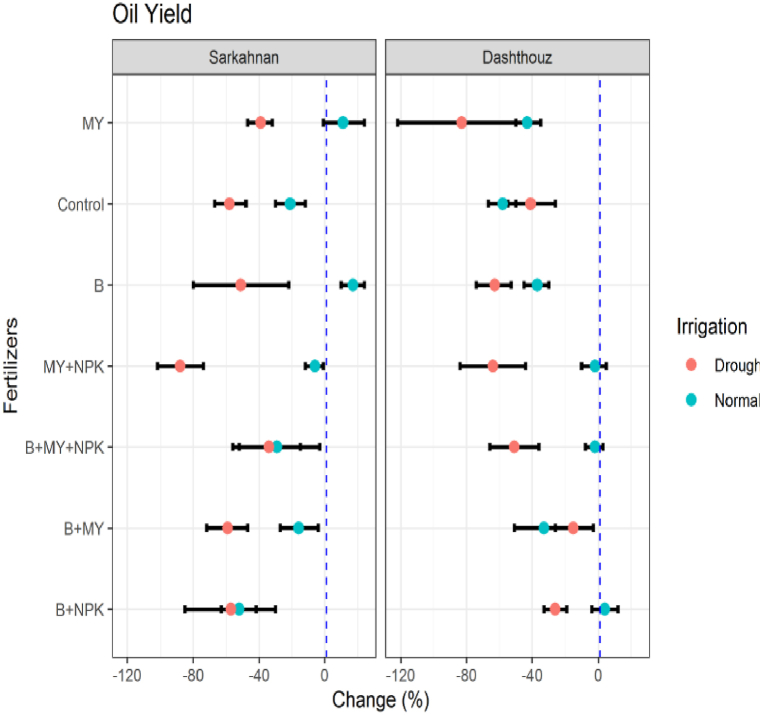
Percentage changes in oil yield in two locations (Dashthouz and Sarkahnan) under drought stress and normal conditions with the application of different types of fertilizers. (the changes are calculated compared to the chemical fertilizer (NPK) treatment).

The oil yield of sesame showed significant variations across different treatments in both locations and irrigation conditions. Among the treatments in Sarkahnan under normal irrigation, the highest increase in oil yield was observed with the B fertilizer treatment, showing a percentage change of 17 %. This was followed by the MY treatment with an increase of 11 %. On the other hand, the B+MY+NPK treatment exhibited the highest decrease in oil yield, with a percentage change of −29 %. Under drought stress in Sarkahnan, the B treatment had the highest increase in oil yield, with a percentage change of 17 %. The MY treatment also showed a moderate increase of 11 %. In contrast, the MY+NPK treatment had the highest decrease in oil yield, with a percentage change of −88 %. Moving to Dashthouz under normal irrigation, the B+NPK treatment displayed the highest increase in oil yield, with a percentage change of 4 %. Meanwhile, the B+MY treatment showed the highest decrease, with a percentage change of −33 % (Fig. 10). Under drought stress in Dashthouz, the B+NPK treatment had the highest increase in oil yield, with a percentage change of −26 %. Conversely, the MY treatment exhibited the highest decrease, with a percentage change of −83 %.

#### 3.1.10 Meal yield

**Fig. 11:**
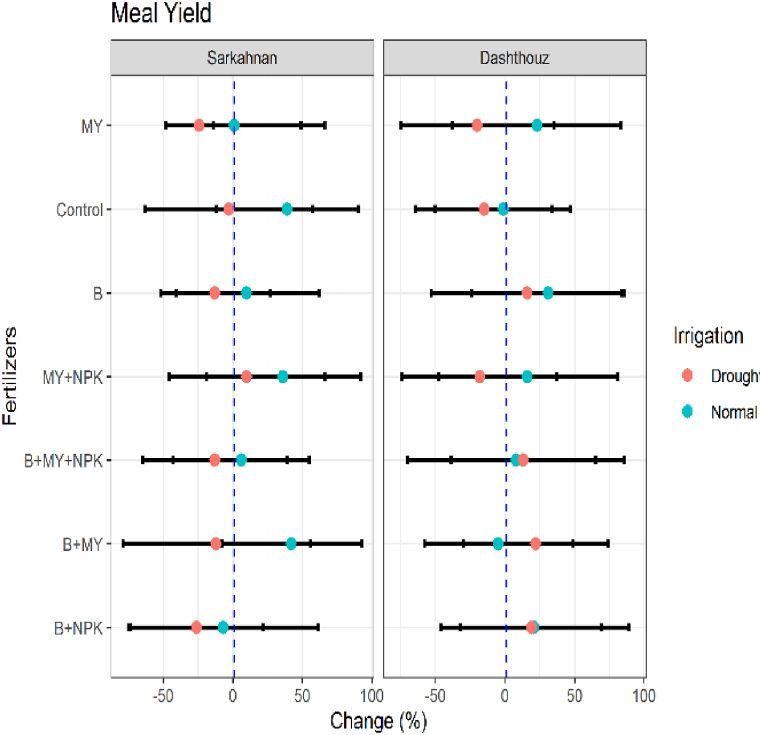
Percentage changes in meal yield in two locations (Dashthouz and Sarkahnan) under drought stress and normal conditions with the application of different types of fertilizers. (the changes are calculated compared to the chemical fertilizer (NPK) treatment).

The meal yield of sesame was evaluated in the study, and the results showed varying percentage changes across different treatments and irrigation conditions. In Sarkahnan under normal irrigation, the B+NPK treatment exhibited the lowest percentage change of −7% in meal yield, while the B+MY treatment showed the highest percentage change of 42 %. Under drought stress in Sarkahnan, the B+NPK treatment had the lowest percentage change of −26 %, while the MY+NPK treatment had the highest percentage change of 10 %. In Dashthouz under normal irrigation, the B+MY treatment had the lowest percentage change of −5%, while the B treatment had the highest percentage change of 31 % in meal yield. Under drought stress in Dashthouz, the MY+NPK treatment had the lowest percentage change of −18 %, while the B+MY treatment showed the highest percentage change of 22 % (Fig. 11).

#### 3.1.11 Water use efficiency

**Fig. 12:**
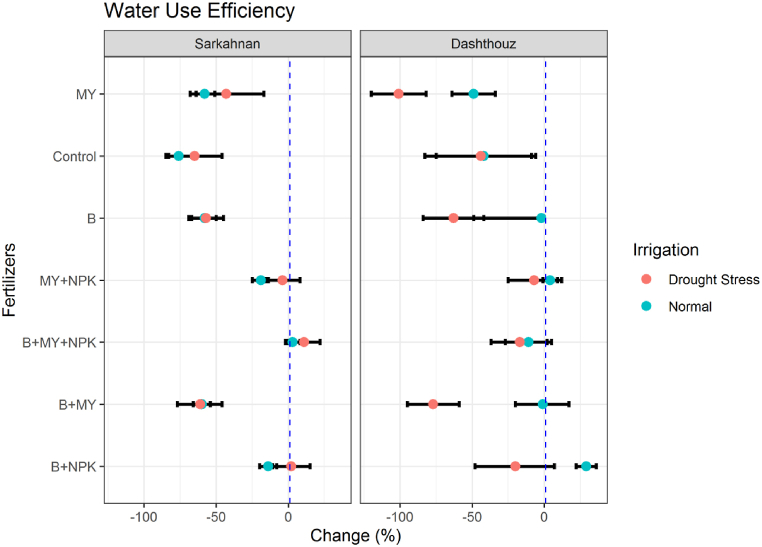
Percentage changes in water use efficiency in two locations (Dashthouz and Sarkahnan) under drought stress and normal conditions with the application of different types of fertilizers. (the changes are calculated compared to the chemical fertilizer (NPK) treatment).

In Sarkahnan under normal irrigation, the lowest percentage change in water use efficiency was observed in the B+MY treatment with a decrease of 60 %. The highest percentage change was seen in the B+MY+NPK treatment with an increase of 3 %. Under drought stress in Sarkahnan, the lowest percentage change in water use efficiency was observed in the B+MY treatment with a decrease of 61 %. The highest percentage change was seen in the B+MY+NPK treatment with an increase of 11 %. In Dashthouz under normal irrigation, the lowest percentage change in water use efficiency was observed in the B+MY treatment with a decrease of 1 %. The highest percentage change was seen in the B+NPK treatment with an increase of 29 % (Fig. 12).

The application of fertilizer treatments had a significant effect on all measured yield components under both normal irrigation and drought stress conditions (p < 0.05). In the context of normal irrigation, the concurrent application of bacteria, mycorrhizal fungi, and NPK (B+MY+NPK) consistently yielded the highest seed yield, biological yield, and water-use efficiency (WUE) across both locations, with a 29 % increase in biological yield and a 17 % improvement in WUE in comparison to NPK alone. Conversely, under conditions of drought stress, treatments incorporating bio-fertilizers (e.g., B+MY and B+MY+NPK) exhibited notable resilience, with yield losses reduced by up to 15 % in comparison to chemical fertilizers alone. For instance, the B+MY treatment enhanced seed yield by 12 % and oil yield by 9 % in comparison with NPK in Sarkahnan. These findings underscore the potential of bio-fertilizers in mitigating the deleterious effects of water scarcity. The results also revealed notable differences between locations. In Dashthouz, the low organic matter and high sand content limited the efficacy of MY-alone treatments, whereas Sarkahnan showed more pronounced benefits from bio-fertilizers due to higher baseline fertility. These location-specific interactions underscore the importance of adapting fertilizer strategies to soil and environmental conditions.

#### 3.1.12 Correlation

**Fig. 13:**
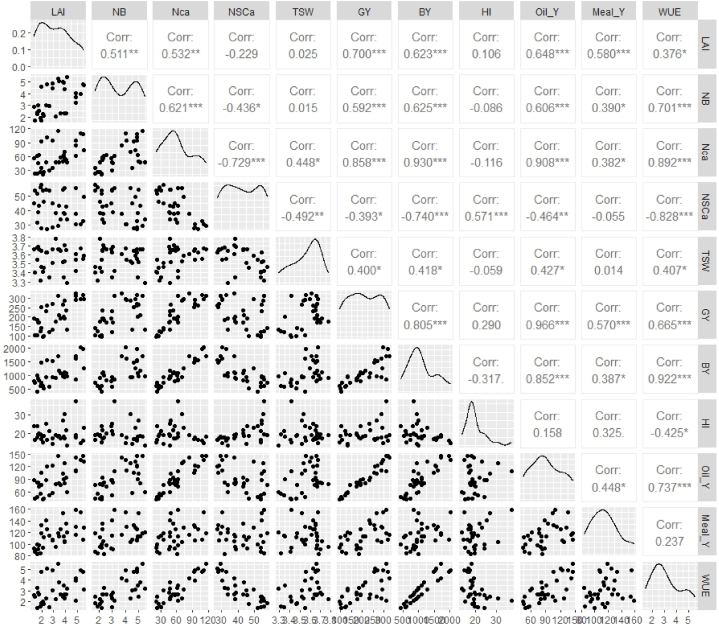
Correlation Analysis of Parameters in Sesame Cultivation: Interrelationships and Implications (In this figure, LAI: Leaf area index, NB: Number of branches, Nca: number of capsules, NSCa: number of seeds per capsules, TSW: thousand seed weight, GY: green yield, BY: Biological yield, HI: Harvest index, Oil_Y: Oil yield, Meal_Y: Meal yield and WUE: Water use efficiency**.**

The figure presents a correlation analysis of various parameters in sesame cultivation, thereby showcasing their correlation. A significant positive correlation is observed between Leaf Area Index (LAI) and Green Yield (GY) (Corr: 0.700∗∗), and between LAI and Biological Yield (BY) (Corr: 0.623∗∗∗). This finding indicates that an enhancement in LAI is associated with an increase in both GY and BY. In a similar vein, a significant correlation is observed between GY and BY (Corr: 0.930∗∗∗) and Oil Yield (Oil_Y) (Corr: 0.966∗∗∗), underscoring their interdependence. Notable negative correlations were observed between the Number of Seeds per Capsule (NSCa) and Water Use Efficiency (WUE) (Corr: −0.828∗∗∗) and Green Yield (Corr: −0.740∗∗∗), suggesting an inverse relationship. Thousand Seed Weight (TSW) has been found to demonstrate significant positive correlations at the 5 % level with both Green Yield (Corr: 0.448∗) and Biomass Yield (Corr: 0.418∗). Harvest Index (HI) has been found to correlate positively with Oil Yield (Corr: 0.852∗∗), but weaker or non-significant relationships have been observed with other traits. Oil Yield is closely related to Meal Yield (Meal_Y) (Corr: 0.737∗∗∗) and WUE (Corr: 0.922∗∗∗), highlighting its central role in determining sesame productivity. Non-significant or weak correlations are observed for TSW and HI with several parameters, implying limited direct influence. The findings emphasise the complexity of trait interactions in sesame cultivation, underscoring the significance of traits such as LAI, GY, and Oil_Y for enhancing yield and efficiency (Fig. 13).

## 4 Discussion

This study emphasises the pivotal function of drought stress and fertilisation treatments in sesame cultivation, underscoring the significance of integrated nutrient management for augmenting crop growth, yield, and efficiency under water-restricted conditions. The research provides valuable insights into how combining chemical fertilizers with biological fertilizers, such as plant growth-promoting rhizobacteria (PGPR) and arbuscular mycorrhizal fungi (AMF), can improve plant resilience and productivity. These findings are of particular significance for advancing sustainable agricultural practices, where reducing the reliance on chemical inputs is essential for both environmental and economic sustainability.

Drought stress has been shown to have a detrimental effect on plant growth, as evidenced by a reduction in leaf area index (LAI), the number of branches, and biological yield [23,24]. This finding is consistent with the observations reported in the studies conducted by Rady et al. [23] and Najafi Vafa et al. [24]. These studies established that water scarcity limits nitrogen uptake, impairs photosynthesis, and reduces stomatal conductance. This ultimately leads to lower biomass production and seed yield. The present study observed analogous reductions in LAI and yield components under drought conditions. However, the use of biological fertilizers s, such as PGPR and AMF, was found to mitigate these deleterious effects, suggesting that integrating biological and chemical fertilizers could be a promising strategy for enhancing crop resilience in water-stressed environments. The positive effects of combining NPK fertilizers with PGPR and AMF are particularly noteworthy. Adeyemi et al. [25] have previously demonstrated that AMF enhances phosphorus uptake and promotes root growth under drought stress, while PGPR enhance drought tolerance through mechanisms such as nitrogen fixation, exopolysaccharide (EPS) production, and modulation of stress-responsive hormones [26]. The present study demonstrates that the integrated application of NPK fertilizers, PGPR, and AMF not only enhances nutrient uptake but also improves water-use efficiency (WUE), thereby contributing to the enhanced performance of sesame plants under normal irrigation conditions. These findings are consistent with the conclusions of Zhou et al. [27], who emphasised that adequate nutrition and supplementary irrigation are crucial for enhancing drought tolerance in crops.

The utilization of bio-fertilizers s, such as PGPR, has garnered considerable attention due to their pivotal function in enhancing soil fertility and fostering the proliferation of beneficial soil microorganism populations. In contrast to the excessive use of chemical fertilizers s, bio-fertilizers reduce dependency on synthetic inputs, thus offering a sustainable alternative that can help mitigate the environmental hazards associated with chemical fertilizers overuse [28]. As demonstrated in this study, the integration of bio-fertilizers with chemical fertilizers enhances soil fertility and increases crop production while also improving the plant's ability to withstand environmental stresses. The use of bio-fertilizers, particularly those containing PGPR strains such as Rhizobium, Azospirillum, and Pseudomonas, has been shown to stimulate plant growth by improving nutrient availability, promoting better root development, and enhancing drought tolerance [29]. Furthermore, PGPR strains such as *Enterobacter cloacae* and *Serratia marcescens* have been shown to solubilise insoluble phosphates and fix nitrogen, thereby providing plants with essential nutrients in a more bioavailable form [30]. The incorporation of AMF into fertilisation regimes has been demonstrated to enhance the plant's nutrient acquisition capacity. AMF play an essential role in phosphate solubilization and improve plant phosphorus uptake, reducing the reliance on chemical phosphorus fertilizers. This is particularly beneficial in nutrient-deficient soils, where chemical fertilizers may be less effective. As demonstrated by Adeyemi et al. [25], AMF enhances nutrient uptake without compromising crop yield, thus contributing to more sustainable agricultural systems. The synergistic effects of combining PGPR with AMF, as observed in this study, are consistent with the growing body of literature supporting the use of biological fertilizers for improving plant growth, drought tolerance, and overall yield performance.

The findings of this study underscore the pivotal role of soil characteristics in dictating the efficacy of disparate fertilisation treatments. In regions such as Sarkohanan, which is characterised by high organic matter content and fixed phosphorus levels, the addition of AMF did not yield the expected benefits, likely due to limited phosphorus availability. Conversely, the nutrient-poor soils in Dashthoz exhibited a superior response to combined biological and chemical treatments, suggesting that the interaction between biological fertilizers and soil nutrient status is a critical factor in maximizing their efficacy. This observation is consistent with the findings of Bhardwaj et al. [15], who noted that biostimulants, when applied to low-nutrient soils, can significantly improve nutrient uptake and enhance soil fertility. The findings emphasise the necessity for customised nutrient management strategies that take into account both soil characteristics and the specific environmental conditions of the region.

Furthermore, the study revealed a significant trade-off between reproductive and vegetative growth under drought stress. The negative correlation between water-use efficiency (WUE) and seed number per capsule suggests that, during periods of water scarcity, plants prioritise survival over reproductive output, a physiological adjustment that is common in many drought-stressed species [31]. This trade-off underscores the intricate physiological responses exhibited by sesame plants in response to water stress, emphasizing the necessity for strategic management practices that achieve a balance between vegetative growth and reproductive success under challenging conditions. Another critical aspect of this study is the effect of bio-fertilizer application on seed yield and oil content. While the application of chemical fertilizers alone resulted in higher seed yields, the combined use of biological fertilizers with minimal chemical input yielded comparable results, thus highlighting the potential of bio-fertilizers to reduce the dependency on chemical fertilizers while maintaining or even improving yield. This finding supports the increasing interest in using bio-fertilizers as a viable alternative to chemical fertilizers, which are known to have long-term detrimental effects on soil health and environmental quality (Sakara, 2020). Furthermore, the observed enhancements in oil yield with integrated nutrient management (B+MY+NPK) are presumably ascribed to superior nutrient availability and water retention, as indicated by Ozkan and Kulak [32]. In conclusion, the integration of biological and chemical fertilizers offers a promising strategy for improving sesame growth and yield under drought stress conditions. By reducing the reliance on chemical fertilizers and enhancing the resilience of crops to environmental stress, bio-fertilizers such as PGPR and AMF provide a sustainable solution for improving crop productivity and soil health. However, further research is needed to optimize the application rates, timing, and specific strains of bacteria and fungi to maximize their potential benefits. The study also emphasises the importance of soil-specific nutrient management, as the effectiveness of biological fertilizers is influenced by soil nutrient content and the interaction between soil microorganisms and plant roots. The findings of this study underscore the necessity for a comprehensive approach to fertilizer management that incorporates biological and chemical inputs, thereby promoting sustainable agricultural practices that can assist in addressing the challenges posed by water scarcity and soil degradation.

Also, The observed percentage changes in yield components highlight the critical role of integrated nutrient management under both normal and drought conditions. The enhanced performance of B+MY+NPK in boosting seed yield and WUE underscores the potential synergy between bio-fertilizers s and chemical inputs. These results are consistent with previous studies that have demonstrated the capacity of Pseudomonas putida and mycorrhizal fungi to enhance nutrient availability and drought tolerance through nitrogen fixation and phosphorus solubilization [25,26]. The location-specific variability in treatment efficacy suggests that soil characteristics play a pivotal role in determining the success of bio-fertilizer interventions. While Sarkahnan's higher organic matter content facilitated better nutrient uptake, the sandier soils in Dashthouz limited the efficacy of phosphorus-solubilizing fungi, emphasizing the need for soil-specific strategies. The findings provide actionable insights for sustainable sesame cultivation, demonstrating how integrating bio-fertilizers can reduce dependency on chemical inputs while enhancing resilience to water scarcity. This has broader implications for addressing challenges in arid and semi-arid regions under climate change.

## 5 Conclusion

The present study investigated the influence of different fertilizers and irrigation conditions on multiple traits in sesame cultivation. The findings demonstrated varied responses across diverse parameters, including leaf area index (LAI), seed count per capsule, thousand seed weight (TSW), biological yield (BY), harvest index (HI), oil yield (Oil_Y), meal yield (Meal_Y), and water use efficiency (WUE). Under standard irrigation conditions, the B+MY treatment demonstrated significant enhancements in LAI and the number of seeds per capsule, suggesting enhanced plant growth and reproductive potential. Furthermore, the B treatment demonstrated significant increases in BY and HI, suggesting enhanced overall crop productivity. The B+MY combination demonstrated the most substantial increase in Oil_Y, suggesting its capacity for achieving elevated oil production. Furthermore, the B+MY+NPK combination exhibited the highest WUE, suggesting efficient water usage in sesame cultivation. However, it is important to note that certain traits displayed varying responses under drought stress conditions. For instance, the B+MY treatment exhibited a substantial increase in the number of seeds per capsule, while the B+MY+NPK treatment demonstrated a significant decrease in this trait. Furthermore, the B treatment demonstrated a significant increase in BY, while the control treatment exhibited a slight decrease in BY under drought stress. These findings underscore the necessity of contemplating bespoke management practices to mitigate the repercussions of drought stress on sesame production. In conclusion, the study provides valuable insights into the selection of appropriate fertilizers and irrigation strategies to optimize sesame cultivation for desired traits. The results emphasise the necessity for customised approaches, contingent on specific trait objectives and environmental conditions. Further research is warranted to validate these findings and explore additional factors that may contribute to sesame yield and quality.

The results of this study emphasise the pivotal function of bio-stimulants, including Pseudomonas putida and arbuscular mycorrhizal fungi (AMF), in augmenting nutrient uptake and water-use efficiency under drought conditions. The mechanisms through which these bio-stimulants facilitate improved nutrient absorption include root symbiosis, increased soil water retention, and the production of growth-promoting substances. These mechanisms enable sesame plants to maintain physiological processes despite water scarcity. The combination of bio-stimulants with chemical fertilizers (B+MY+NPK) exhibited a particularly pronounced synergistic effect in mitigating the deleterious effects of drought on crop productivity. The results of this study highlight the potential of integrating bio-stimulants into sustainable agricultural practices to reduce reliance on chemical inputs while enhancing crop resilience to drought stress. Future research should focus on optimizing the application rates and combinations of bio-stimulants to enhance their effectiveness across diverse environmental conditions.

## CRediT authorship contribution statement

**Nasser Nourzadeh:** Visualization, Validation, Resources, Project administration, Methodology, Investigation. **Asghar Rahimi:** Project administration, Methodology, Investigation,Writing – review & editing. **Amir Dadrasi:** Writing – review & editing, Writing – original draft, Formal analysis, Data curation.

## Data/code availability statement

Data will be made available on request. For requesting data, please write to the corresponding author.

## Declaration of competing interest

The authors declare that they have no known competing financial interests or personal relationships that could have appeared to influence the work reported in this paper.
